# Awards and Criticisms

**Published:** 1889-08-03

**Authors:** 


### THE METROPOLITAN HOSPITAL SUNDAY FUND, 1889.

### AWARDS AND CRITICISM.

THE annual general report of the Committee of Distribution, which was adopted by the Council of the Metropolitan Hospital Sunday Fund on the 29th July, is remarkable because it declines, after full and careful investigation, to give an award to either the St. John's Hospital for Diseases of the Skin, or to the West End Hospital for Diseases of the Nervous System. The report is as follows:

"Your committee have the honour to recommend the payment of awards to the 161 institutions enumerated in the appendix to this report, being three more than last year, and an increase of 56 since the first awards were made in 1873.

"The total amount available for distribution, after allowing sufficient for liabilities and the usual current expenses, is £40,746. Of this total £38,481 5s. is now recommended to 111 hospitals and 50 dispensaries.

"Five per cent. of the total collected—viz., £2,000—is set apart to purchase surgical appliances, in obedience to law xii., in monthly proportions during the ensuing year.

"Your committee recommend that all payments to the fund after this date be carried to the credit of next year's fund.

"In compliance with an order of the council, and for the special use of its members, tables of statistics have been prepared as usual, showing an analysis of the number of beds in hospitals, the cost of patients both at hospitals and dispensaries, the proportionate expense of management, as well as other valuable information, and copies are now sent to every member of the council.

"The number of deputations representing committees of various hospitals, invited to confer with your committee, and to offer explanations on matters of apparently unsatisfactory character, was only nine this year as compared with eleven in 1888. Four of these deputations attended, and in two cases your committee regret to report that, after these interviews, they are unable to recommend any award. Five institutions sent replies, but did not attend. The application from one dispensary was withdrawn.

"Two institutions which have been less than three years established, and hence could not comply with law iv. by furnishing balance-sheets for three years, had to be refused. One application, from a nursing institution, which, being neither hospital nor dispensary, could not be entertained; and two from other institutions came too late.

"Mansion House, 23rd July, 1889."

